# The Emerging Role of SPECT Functional Neuroimaging in Schizophrenia and Depression

**DOI:** 10.3389/fpsyt.2021.716600

**Published:** 2021-12-15

**Authors:** Anil Kalyoncu, Ali Saffet Gonul

**Affiliations:** Department of Psychiatry, Ege University School of Medicine, Izmir, Turkey

**Keywords:** depression, SPECT, dopamine, schizophrenia, molecular imaging

## Abstract

Over the last three decades, the brain's functional and structural imaging has become more prevalent in psychiatric research and clinical application. A substantial amount of psychiatric research is based on neuroimaging studies that aim to illuminate neural mechanisms underlying psychiatric disorders. Single-photon emission computed tomography (SPECT) is one of those developing brain imaging techniques among various neuroimaging technologies. Compared to PET, SPECT imaging is easy, less expensive, and practical for radioligand use. Current technologies increased the spatial accuracy of SPECT findings by combining the functional SPECT images with CT images. The radioligands bind to receptors such as 5-hydroxytryptamine 2A, and dopamine transporters can help us comprehend neural mechanisms of psychiatric disorders based on neurochemicals. This mini-review focuses on the SPECT-based neuroimaging approach to psychiatric disorders such as schizophrenia and major depressive disorder (MDD). Research-based SPECT findings of psychiatric disorders indicate that there are notable changes in biochemical components in certain disorders. Even though many studies support that SPECT can be used in psychiatric clinical practice, we still only use subjective diagnostic criteria such as the Diagnostic Statistical Manual of Mental Disorders (DSM-5). Glimpsing into the brain's biochemical world via SPECT in psychiatric disorders provides more information about the pathophysiology and future implication of neuroimaging techniques.

## Introduction

Diagnosis of psychiatric disorders has always been debated, yet there is still no objective diagnostic tool. Throughout the diagnostic revision process of psychiatric diseases, schizophrenia, and depression are two of the most valid and stable psychiatric disorders regarding diagnostic criteria. However, even though diagnostic criteria have evolved to diagnose these diseases better, we still lack the insight to comprehend the biological aspects of these disorders. Thus, there is a huge gap in diagnosis and providing biological information for these disorders via neuroimaging methods.

Functional and structural imaging methods have contributed to a better understanding beyond the molecular process of psychiatric disorders (1). Magnetic resonance imaging (MRI), functional magnetic resonance imaging (fMRI), SPECT, and positron emission tomography (PET) have evolved to enlighten complex psychiatric disorders. Among these imaging methods, SPECT and PET have similar properties in terms of methods they use. Basically, both methods give information based on the spatial concentration of injected radiopharmaceuticals. The fundamental difference between PET and SPECT stems from the radiotracers they utilize. While SPECT uses heavy isotopes like ^99m^Tc and ^123^I that emit gamma-ray, PET uses lighter isotopes such as ^11^C, ^13^N, and ^18^F that emit positrons. The half-life of the pharmaceutical used in SPECT is longer than PET, which makes it possible to perform more longitudinal scans. The cost of SPECT is lower than PET which makes it much easier to use in clinical practice and more accessible. Despite the many advantages of SPECT imaging, PET offers more spatial resolution than SPECT (2).

Radiotracers used in brain imaging should have some features for obtaining high-quality images. These are high affinity, specificity for the desired target, low non-specific binding, low plasma protein binding, ability to pass through the blood-brain barrier, and high plasma clearance (3). The most used radiotracer in Brain SPECT is Tc-99m-hexamethylpropylene amine oxime (HMPAO) which shows perfusion. Perfusion SPECT can be used to diagnose and assess neuropsychiatric pathologies such as dementia, traumatic brain injury (TBI), toxin exposure, and inflammatory disorders by detecting hypoperfusion in the brain (4, 5). Moreover, perfusion SPECT gives us essential clues in many psychiatric disorders. Perfusion SPECT can help estimate typical stimulant medication response in children with attention deficiency and hyperactivity disorder (ADHD) (6). It can show blood perfusion alteration in treatment resistant MDD (7). Many SPECT studies regarding psychiatric disorders are based on perfusion, but neurochemical SPECT studies provide more specific information about the pathophysiology. That target-specific feature makes SPECT a valuable tool for psychiatric research.

In this mini-review, we summarized and discussed SPECT findings of schizophrenia and depression. To investigate SPECT studies in patients with schizophrenia and depression, we performed a systematic literature search on PubMed database using the keywords “SPECT,” “Schizophrenia,” and “Depression” and any of following words: “GABA,” “Serotonin,” “Glutamate,” and “Dopamine.” Most relevant and recent studies on the subject are included in this mini-review in consideration of the balance between developments and limitations in this area.

### SPECT in Schizophrenia

**Figure 1:**
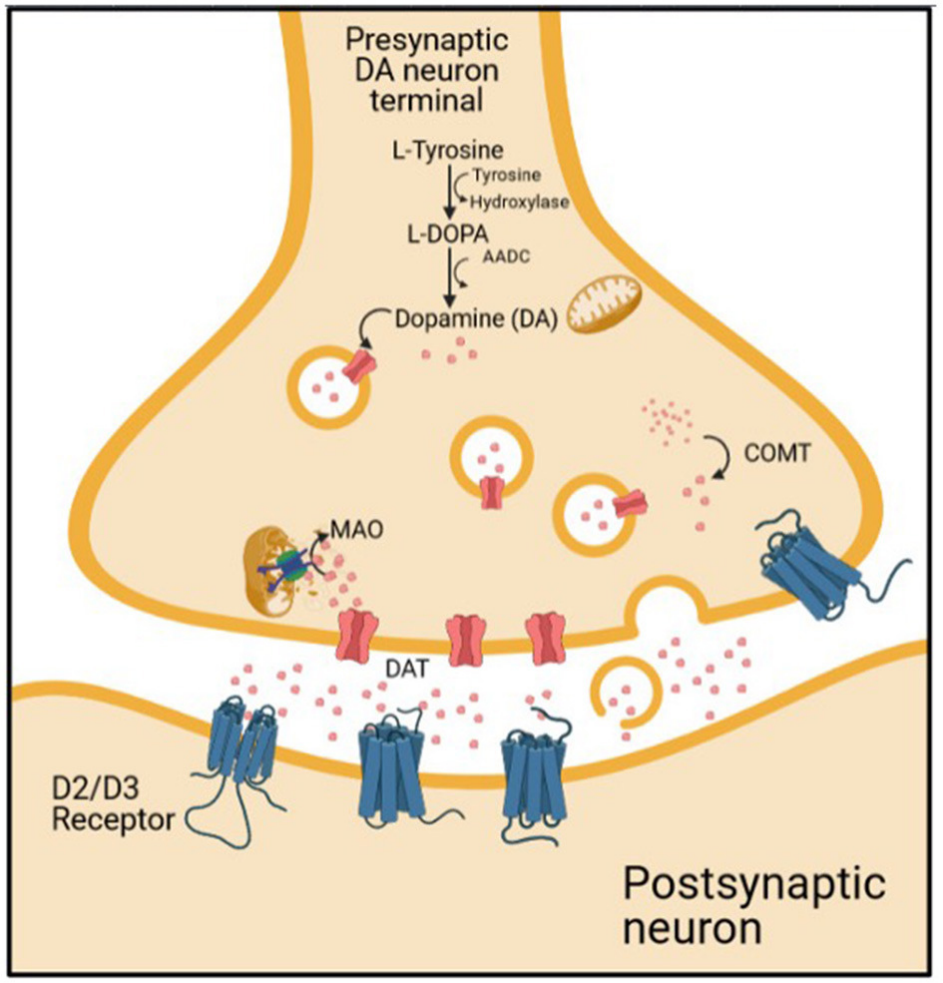
The dopaminergic transmission on synaptic cleft.

Schizophrenia is a heterogenous and chronic psychiatric disorder that manifests with positive and negative symptoms. These symptoms arise from complex molecular alterations in the brain. The molecular aspect of schizophrenia has been investigated for many years to understand the nature of the disease better. One of the most studied molecular components of schizophrenia is dopamine (8). Dopaminergic pathways consist of presynaptic and post-synaptic compartments. The presynaptic compartment comprises dopamine synthesis, dopamine storage into vesicle by vesicular monoamine transporter 2 (VMAT), dopamine degradation by monoamine oxidase (MAO), dopamine release, and dopamine reuptake by dopamine transporter (DAT). The post-synaptic compartment includes dopamine receptors and post-receptor signaling [(9); Figure 1].

### Imaging of Dopaminergic System

It has been previously known that dopaminergic pathway alteration takes part in schizophrenia pathophysiology. Many studies with imaging methods indicate that increased subcortical dopamine activity and decreased cortical dopamine are part of the disease process (10). Of those imaging tools that are targeting the molecular mechanism of schizophrenia, SPECT has a significant contribution. The first SPECT study on schizophrenia was published in 1986 (11). ^77^Br-bromospiperone, which is not popular today due to poor imaging characteristics, was used in that study to show D2 receptor density.

With the advancement of dopamine studies in schizophrenia, new molecules have emerged to assess post-synaptic dopaminergic activity, such as [^123^I] iodobenzamide (IBZM) and [^123^I] epidepride [(12); Table 1]. These radiotracers act by binding to the post-synaptic receptors and allow us to assess post-synaptic dopamine activity. However, antipsychotics used for the treatment of schizophrenia also bind to the same receptor and display the radiotracer. Thus, patients taking antipsychotic medications may test at lower levels of dopamine receptor availability than is actually the case (28). In studies on the effects of antipsychotics on dopamine receptors, the basal ganglia/frontal cortex ratio, which shows striatal dopaminergic activity, was found to be negatively correlated with extrapyramidal side effects (13, 14). In addition, the BG/FC ratio of IBZM was lower in inadequate treatment response to antipsychotics (14). In general, in meta-analysis studies, we see that the post-synaptic D2/D3 receptor activity of patients with schizophrenia is slightly elevated, but this effect is not significant in patients who do not receive treatment (16, 29).

**Table 1:** Binding sites and summary of studies of available SPECT radiotracers for dopamine, serotonin, GABA, and glutamate.

**Radiotracers**	**Neurotransmitters**	**Binding sites**	**Additional findings**
[^123^I] iodobenzamide	Dopamine	Striatal post-synaptic D_2_/D_3_ receptor	BG/FC binding ratio was found to be negatively correlated with EPS and lower in patients with schizophrenia on antipsychotic treatment (13, 14).
[^123^I] epidepride	Dopamine	Extrastriatal frontal D_2_/D_3_ receptors	Frontal D_2_/D_3_ binding was found to be positively correlated with positive symptom reduction (15).
[^123^I]-FP-CIT	Dopamine, serotonin	DAT, SERT	No significant difference was found between schizophrenia and HC in a meta-analysis study (16).
[^123^I]-β-CIT	Dopamine, serotonin	DAT, SERT, NET	No significant difference was found between schizophrenia and HC in a meta-analysis study (16).
[^123^I] nor-β-CIT	Dopamine, serotonin	DAT, SERT, NET	No significant difference was found between schizophrenia and HC in a meta-analysis study (16).
[^99m^Tc]-TRODAT	Dopamine	DAT	No significant difference was found between schizophrenia and HC in a meta-analysis study (16).
[^123^I] iomazenil	GABA	GABA_A_ receptors (α1, α2, α3, and α6 subunits)	Inconsistent results have been reported in patients with schizophrenia (17).
[^123^I] CNS-1261	Glutamate	NMDA receptor	Global binding was found to be reduced in schizophrenia on clozapine treatment. No significant difference was found between HC and treatment naïve schizophrenia (18).
[^123^I] ADAM	Serotonin	SERT	Results are controversial. Some studies found no significant difference between HC and depression (19–21). Other studies found lower SERT bioavailability in depression (22–24).
[^123^I] R91150	Serotonin	5HT2A	Compared to equivalent PET tracers, it has a lower signal-to-noise ratio. Harm avoidance was found correlated with high DLPFC 5HT2A binding (25–27).

Another process of the dopaminergic pathway, which is dopamine release into the synaptic cleft, could also be probed *in vivo* by IBZM (30). Dopamine release into the synaptic cleft could be assessed by the combination of dopamine release induction by amphetamine or methylphenidate and SPECT technique. When the dopamine receptors are occupied by dopamine, it results in decreased radiotracer binding. There is a negative linear relationship between dopamine release and radiotracer availability. Imaging studies assessing dopamine release in patients with schizophrenia indicate an increased amphetamine-induced dopamine release (31–33).

[^123^I] epidepride is another radiotracer to identify extrastriatal frontal D_2_/D_3_ receptors, which involve cognitive functions like planning, attention, and task switching (34–36). Inhibition of these extrastriatal receptors by antipsychotics results in decreased attentional focus, as shown in a previous study (15). Moreover, positive symptom reduction was positively correlated with the binding potential of frontal D_2_/D_3_ receptors in the three mounts of zuclopenthixol and risperidone treatment study (15).

The dopamine transporter is another part of the dopaminergic system. SPECT studies comparing schizophrenia with healthy controls (HC) that assess dopamine transporter by [^123^I]-FP-CIT (N-omega-fluoropropyl-2beta-carboxymethoxy-3beta-{4-iodophenyl} tropane), [^123^I]-β-CIT (Iodine-123-beta-carbomethoxy-3 beta-(4-iodophenyltropane)), and [^99m^Tc]-TRODAT show no difference (16). Besides the dopamine transport and post-synaptic dopamine receptor, dopamine synthesis is one of the important processes of the dopamine pathway in schizophrenia. Even though SPECT studies cannot address it, PET meta-analysis shows elevated dopamine synthesis capacity in schizophrenia (16).

It could be inferred from these findings that the synaptic space of the dopaminergic system might be affected by the treatment process rather than by the disease itself (29). Studies related to the presynaptic dopaminergic system, mainly investigated by PET rather than SPECT due to lack of suitable radiotracers, point out that increased dopamine release and dopamine synthesis capacity result in presynaptic dopaminergic neurotransmission activity (37). This presynaptic dopaminergic activity is regulated primarily by other neurotransmitter pathways such as glutamate and GABA (28).

### Imaging of GABAergic System

GABA has an essential role in the molecular aspects of schizophrenia pathogenesis by regulating presynaptic dopaminergic activity (28, 38). *In vivo* imaging and post-mortem studies evaluating the role of GABA in schizophrenia show important results. SPECT studies can visualize α1, α2, α3, and α6 subunits of GABA_A_ receptors by utilizing [^123^I] iomazenil (39). Inconsistent results were reported in different studies evaluating GABA bioavailability and symptom severity in schizophrenia. While no correlation was found in two of the studies investigating the relationship between symptom severity and GABA receptor availability in patients with schizophrenia (40, 41), a correlation was found in one study evaluating this relationship based on negative and positive symptoms (42). Negative symptoms were found to be negatively correlated with the GABA binding in medial frontal region, whereas positive symptoms were found to be negatively correlated with GABA binding in the medial temporal lobe (42). Three voxel-wise studies to evaluate regional differences in the brain show that GABA binding is generally low despite regional inconsistencies (40, 43, 44).

Even though there are inconsistent results in GABA studies in schizophrenia, the most replicated findings are reduced glutamic acid decarboxylase 67 (GAD67) mRNA, which plays a role in cytosolic GABA-synthesis, in post-mortem studies (45–47). Subtypes of alpha unit findings in post-mortem studies show that there is a decrease in alpha 1 (47–49) subunit and an increase in alpha 2 subunit (49, 50), and inconsistent results for alpha 5 subunit (50–52).

A systematic review comparing HC and schizophrenia regarding GABA_A_/BZ receptor binding availability reported no significant group differences (17). Consequently, even consistent results have been replicated in post-mortem studies and *in vivo* neuroimaging studies have not shown promising results for GABA alteration in patients with schizophrenia.

### Imaging of Glutamatergic System

Dysfunction in GABA results in disinhibition of the glutamatergic pathway and asynchronous cortical activity (17). Glutamate acts as a regulatory neurotransmitter in the presynaptic dopaminergic pathway as well as GABA. Glutamate has both G-coupled metabotropic and ligand-gated ionotropic receptors. It has been demonstrated that NMDA, which is an ion-gated glutamate receptor, is involved in schizophrenia pathogenesis (53). However, SPECT studies that investigate NMDA levels are insufficient despite the importance of glutamatergic transmission in schizophrenia. In literature, the glutamatergic pathway has been assessed by [^123^I] CNS-1261. [^123^I] CNS-1261 acts as an NMDA receptor ligand. One SPECT study indicates that the total volume binding distribution of [^123^I] CNS-1261 does not show any difference between healthy control and drug-free schizophrenia patients (18). In the same study, global binding of [^123^I] CNS-1261 was reduced in patients on clozapine treatment compared with drug-free patients (18). This result could be related to downregulation due to clozapine treatment, disease-related pathology, or competition of clozapine and radiotracer at the binding site. Further study revealed a significant negative correlation between [^123^I] CNS-1261 binding and residual symptom severity in patients under treatment of typical antipsychotics (18). Otherwise, the symptom duration of the medication-free group was positively correlated with [^123^I] CNS-1261 binding in the middle inferior frontal cortex (54). These two studies may underpin the probable importance of the glutamatergic system in schizophrenia.

### SPECT in Depression

Depression is a widespread disabling mental disorder that lifetime prevalence is around 20%, with a high risk of recurrence (55). Various molecular pathologic processes lie behind depression, which have not been fully comprehended. Molecular imaging of depression has been a widely investigated field to establish molecular aspects of depression. One of the most prominent suggestions to elucidate this brain disorder is the monoamine hypothesis which proclaims that depressive patients have lower serotonin, noradrenaline, and dopamine (56). This argument has been studied for the last decades, and the research put forward some evidence on that subject. However, the monoamine hypothesis is still debated, and there is still insufficient evidence of this subject (57). Molecular imaging methods may help with this controversial topic via advanced radiotracers to assess dopamine, noradrenalin, and serotonin levels, and, lately, the degrading enzyme of these monoamines called monoamine oxidase (MAO). Unfortunately, MAO cannot be assessed by SPECT imaging due to lack of radiotracer.

### Studies of Blood Perfusion

Although neuroimaging studies focus on brain function and cerebral blood flow, in this review, we will largely discourse the molecular imaging of neurotransmitters and receptors in major depressive disorder. Briefly, the articles have reported hypoactivity in dorsolateral prefrontal cortex, pregenual anterior cingulate cortex, posterior anterior cingulate cortex, left superior temporal gyrus, insula, and cerebellum (58). In contrast, the subcortical structures (caudate, thalamus) and limbic structures (amygdala, anterior hippocampus) show hyperactivity (58).

One of the other significant regional activity findings related to depression is subgenual anterior cingulate cortex (sgACC) hyperactivity, which has been shown in studies that hyperactivity of sgACC is related to treatment-resistant depression (59, 60). The relationship between sgACC activity and treatment response has been studied many times, and this finding could be beneficial for assessing treatment response in clinical practice in the future.

### Imaging of Serotoninergic System

In addition to cerebral blood flow imaging studies, neurochemical imaging of depression provides information on molecular alteration. The most known neurotransmitter in depression is serotonin. The serotoninergic system is responsible for regulating sleep, stress responses, pain, motor activity, cognition, emotional behavior, appetite, aggression, and impulsivity which are prominently altered in depression (61). However, it is still not well-known how the serotoninergic system is impaired in depression.

Serotonin is synthesized from tryptophan which is transported by large amino acid transporter over the blood-brain barrier. First, tryptophan is hydroxylated by tryptophan hydroxylase to 5-hydroxy-tryptophan, and then 5-hydroxy-tryptophan is decarboxylated into 5-hydroxy-tryptamine (5-HT), which is known as serotonin (62). Then, the produced serotonin is stored into synaptic vesicles by a vesicular monoamine transporter. Vesicles fuse with the synaptic membrane, and serotonin is secreted into the synaptic cleft. After serotonin is released into the synaptic cleft, it binds to its specific receptor, crucial for post-synaptic transmission. The effect of synaptic transmission is resolved by serotonin reuptake via serotonin transporter (SERT) (62).

Another essential structure related to the serotonergic system in depression is the serotonin receptors, which have various subtypes, including 5HT1A, 5HT1B, and 5HT2A. 5HT1A receptors are located in the presynaptic compartment of serotoninergic cell bodies of raphe nucleus and post-synaptic side of terminal area. The 5HT1A receptors are Gi-coupled receptors that hinder neuronal transmission (63). [^123^I] p-MPPI (4-(2′-methoxy-) phenyl-1-[2′-(N-2′′-pyridinyl)-p-iodobenzamido-]ethyl-piperazine) is a SPECT radiotracer that binds 5HT1A receptors *in vivo* which is available for rat and non-human primate but not for human. 5HT1A imaging via SPECT radioligands is restricted due to possible brain excretion by efflux transporter (25). 5HT1B is the other receptor on serotoninergic neuron terminal, an autoreceptor that regulates 5-HT levels by downregulating the serotoninergic system (63). To investigate this molecular process, SPECT provides an opportunity to assess the serotoninergic system. However, evaluation of serotonin synthesis by SPECT is currently limited with SERT and 5HT2A receptors in humans due to the lack of suitable radiotracers.

### Imaging of SERT

The SERT bioavailability is assessed by [^123^I] β-CIT and its analog [^123^I] nor-β-CIT, which has a 10-fold higher affinity to SERT than [^123^I] β-CIT (62, 64, 65). These two radiotracers are not specifically binding to SERT but also bind to noradrenaline transporter (NET) and dopamine transporter (DAT) (66). [^123^I] ADAM is a recently developed radiotracer for SPECT, and it is more specific to SERT than [^123^I] β-CIT and [^123^I] nor-β-CIT (67). However, it is still controversial what SERT availability represents. The increased availability of SERT might indicate enhanced serotonin clearance in the synaptic cleft and vice versa (68). On the other hand, the low level of endogenous serotonin might also impact SERT availability by downregulating it (69, 70). In consequence of unclear alteration of SERT bioavailability in depression, SPECT studies assessing SERT bioavailability in depression have inconsistent results.

The serotonin transporter enriched regions are midbrain, thalamus/diencephalon, and medial prefrontal cortex (mPFC) (71). Some SPECT studies comparing SERT availability in the midbrain between healthy controls and depressive patients show no difference (19–21). However, some studies also indicate that SERT binding is decreased in the midbrain (22–24). The SERT density in the midbrain might be negatively correlated with depression severity (24). Perceived adverse life events, which is considered a trigger of depression, impact SERT by reducing it even in healthy subjects (72). Moreover, one study asserts that higher midbrain SERT is related to antidepressant treatment efficiency (73). During treatment with selective serotonin reuptake inhibitors (SSRI), higher occupancy of SERT by SSRI is correlated with a lower Hamilton Depression Rating Scale Score (74). A recent study with drug-naïve first episode major depressive disorder (MDD) patients found no SERT bioavailability difference between HC and MDD, but found a positive correlation between SERT bioavailability and kynurenine/tryptophan ratio, which indicates tryptophan metabolism (75). A meta-analysis of *in vivo* SERT imaging studies found significantly reduced SERT availability in striatum, amygdala, and brainstem but no significant change in thalamus and hippocampus in *in vivo* studies. In addition, a meta-analysis of post-mortem studies did not find any SERT alteration in brainstem, frontal cortex, and hippocampus but found a significant reduction in amygdala and striatum (76). These inconsistent findings could be caused by different imaging methods, possible brain atrophy that influences the SERT quantification, and the strength of the studies (75).

### Imaging of 5HT2A

Although there are few SPECT studies associated with 5HT2A receptors in depressed patients, [^123^I] R91150, which better evaluates 5HT2A receptors than their PET equivalents because of their lower signal-to-noise ratio, provides essential information about depression (25–27). [^123^I] R91150 studies are concerned with behavioral patterns in depression rather than directly reflecting the depressive scores. For example, one study evaluating the correlation between harm avoidance and 5HT2A receptors reported that high harm avoidance scores related to shyness, fearfulness, and fatigue are positively correlated with left DLPFC 5HT2A receptor binding (26). Another study of six mounts drug-free patients with suicide attempt reports reduced 5HT2A binding in the frontal cortex (27).

### Conclusion

Many tools have provided advances in the knowledge of molecular aspects of psychiatric disorders. Significant contributors to this progress are molecular imaging methods, including SPECT and PET. Among those imaging methods, SPECT is remarkable for its feasibility and cheapness, which makes it useful in clinical practice (2). However, even though studies support SPECT usage in psychiatric disorders such as evaluating dementia, inflammation, toxic exposure, and TBI, there is still a lack of proper objective diagnostic tools besides the Diagnostic Statistical Manual of Mental Disorders (DSM-5), which is based on subjective criteria (5, 6, 77).

In addition, these subjective criteria address neither the pathophysiology nor the treatment of psychiatric disorders (77). As a result, the pathogenesis, correlation to neurological function, and treatment options of psychiatric disorders with profound morbidity, such as schizophrenia and depression, remain hotly debated. The fact that 60% of DSM-5 diagnoses lose their validity when tested in clinical studies supports this mismatch between DSM-5 diagnostic constructs and the neurobiology of patients (78). In view of the above, we can conclude that new diagnostic methodological approaches, including molecular imaging, are essential to elucidate the molecular pathophysiology underlying these complex psychiatric disorders.

Most of these SPECT studies have a small number of participants. Hence, significant but slight alterations could not be detected. Multi-centered and meta-analytic studies could reveal undetected pathophysiological changes. Available SPECT radiotracers to investigate the neurotransmission process in human is still insufficient. Novel radiotracers may help us obtain detailed information about neurochemical aspects of neurotransmission in psychiatric disorders. Furthermore, schizophrenia and depression have different subtypes and heterogeneity. Combined studies of structural and functional MRI with SPECT might clarify this complexity derived by heterogeneity. Additionally, the machine-learning approach in molecular imaging could help the diagnostic process (79). Further studies using novel radiotracers, combined imaging techniques, and machine-learning algorithms are needed to understand psychiatric disorders better and support the clinical utility of SPECT in the future.

## Author Contributions

AK took the lead in writing the manuscript. AG provided critical feedback, supervision, and helped shape the manuscript. Both authors contributed to the article and approved the submitted version.

## Conflict of Interest

The authors declare that the research was conducted in the absence of any commercial or financial relationships that could be construed as a potential conflict of interest.

## Publisher's Note

All claims expressed in this article are solely those of the authors and do not necessarily represent those of their affiliated organizations, or those of the publisher, the editors and the reviewers. Any product that may be evaluated in this article, or claim that may be made by its manufacturer, is not guaranteed or endorsed by the publisher.
